# Combination therapy with mTOR kinase inhibitor and dasatinib as a novel therapeutic strategy for vestibular schwannoma

**DOI:** 10.1038/s41598-020-60156-6

**Published:** 2020-03-06

**Authors:** Jessica E. Sagers, Roberta L. Beauchamp, Yanling Zhang, Sasa Vasilijic, Limeng Wu, Patrick DeSouza, Richard Seist, Wenjianlong Zhou, Lei Xu, Vijaya Ramesh, Konstantina M. Stankovic

**Affiliations:** 1 Eaton-Peabody Laboratories and Department of Otolaryngology – Head and Neck Surgery, Massachusetts Eye and Ear and Harvard Medical School, Boston, MA 02114 USA; 2 Program in Speech and Hearing Bioscience and Technology, Harvard Medical School, Boston, MA 02115 USA; 3 Harvard Program in Therapeutic Science, Harvard Medical School, Boston, MA 02115 USA; 4 Center for Genomic Medicine, Massachusetts General Hospital and Harvard Medical School, Boston, MA 02114 USA; 5 Edwin L. Steele Laboratories, Department of Radiation Oncology, Massachusetts General Hospital and Harvard Medical School, Boston, MA 02114 USA; 6 Department of Neurology, Massachusetts General Hospital and Harvard Medical School, Boston, MA 02114 USA; 7 Present Address: Cancer Center, Union Hospital, Tongji Medical College, Huazhong University of Science and Technology, Wuhan, Hubei 430023 China; 8 Present Address: Department of Oral and Maxillofacial Surgery, Xiangya Hospital, Central South University, Changsha, Hunan 410008 China

**Keywords:** Tumour-suppressor proteins, Pharmacology

## Abstract

Neurofibromatosis type 2 (NF2) is an inherited disorder characterized by bilateral vestibular schwannomas (VS) that arise from neoplastic Schwann cells (SCs). NF2-associated VSs are often accompanied by meningioma (MN), and the majority of NF2 patients show loss of the *NF2* tumor suppressor. mTORC1 and mTORC2-specific serum/glucocorticoid-regulated kinase 1 (SGK1) are constitutively activated in MN with loss of *NF2*. In a recent high-throughput kinome screen in *NF2*-null human arachnoidal and meningioma cells, we showed activation of EPH RTKs, c-KIT, and SFK members independent of mTORC1/2 activation. Subsequently, we demonstrated *in vitro* and *in vivo* efficacy of combination therapy with the dual mTORC1/2 inhibitor AZD2014 and the multi-kinase inhibitor dasatinib. For these reasons, we investigated activated mTORC1/2 and EPH receptor-mediated signaling in sporadic and NF2-associated VS. Using primary human VS cells and a mouse allograft model of schwannoma, we evaluated the dual mTORC1/2 inhibitor AZD2014 and the tyrosine kinase inhibitor dasatinib as monotherapies and in combination. Escalating dose-response experiments on primary VS cells grown from 15 human tumors show that combination therapy with AZD2014 and dasatinib is more effective at reducing metabolic activity than either drug alone and exhibits a therapeutic effect at a physiologically reasonable concentration (~0.1 µM). *In vivo*, while AZD2014 and dasatinib each inhibit tumor growth alone, the effect of combination therapy exceeds that of either drug. Co-targeting the mTOR and EPH receptor pathways with these or similar compounds may constitute a novel therapeutic strategy for VS, a condition for which there is no FDA-approved pharmacotherapy.

## Introduction

Vestibular schwannoma (VS) is the most common tumor of the cerebellopontine angle and the fourth most common intracranial tumor in humans. VSs are formed from neoplastic Schwann cells of the vestibular nerve and can arise sporadically or as part of a debilitating tumor syndrome known as neurofibromatosis type 2 (NF2) that can include multiple schwannomas, meningiomas, and ependymomas^1^. The majority of VS patients generally report sensorineural hearing loss and tinnitus, with others experiencing dizziness, loss of balance, or facial nerve paralysis. Though VSs are histologically benign tumors (WHO grade I), if left unchecked, they can grow to dangerous size and compress the brainstem, which can lead to death. There is no FDA-approved drug therapy for VS or NF2. Options for management include watchful waiting, surgical intervention to remove the tumor, or radiotherapy to prevent further growth. The latter two options can lead to devastating complications such as facial paralysis and total deafness. Radiotherapy has also been shown to increase the risk of VS malignancy, especially for NF2-associated tumors^2–4^. Therefore, development of non-invasive medical therapies to keep VS growth in check represents a major unmet medical need. No effort to identify a therapeutically useful and well tolerated drug has been fully successful, though recent efforts to reframe the problem of drug development for VS and NF2 are beginning to show more promising results *in vitro*^5–8^.

The vast majority of sporadically arising VSs and all NF2-associated VSs demonstrate inactivating mutations of the *NF2* gene, which encodes the tumor suppressor protein merlin (moesin-ezrin-radixin-like protein, OMIM 607379). Merlin is a cytoskeletal linker protein and member of the ERM (ezrin, radixin, moesin) family that is thought to inhibit tumor growth via contact-dependent growth inhibition, decreased proliferation, and increased apoptosis^9^. Loss of merlin leads to the abnormal activation of an array of mitogenic signaling cascades that normally mediate cell adhesion, cell size, proliferation, motility, morphology, and survival. Key signaling pathways known to become deregulated following loss of merlin include hippo-YAP^10^, Ras/Rac^11^, cMET^12^, EGFR^13^, CD44^14^, mTORC1/2^15–17^, and receptor tyrosine kinases (RTKs)^18^. Clinical trials repurposing FDA-approved drugs targeting these signaling pathways, such as lapatinib for EGFR inhibition^19^ and everolimus for mTORC1 inhibition^20^, have been met with lukewarm success.

The protein kinase complexes containing mTOR (‘mechanistic target of rapamycin’), mTORC1 and mTORC2, direct numerous vital processes relevant to cell growth and proliferation and are often dysregulated in human tumors. Mutations in key proteins integral to signaling pathways upstream of mTORC1/2, such as PI3K, p53, and PTEN, can promote mTOR complex activation and are known to play a role in many genetic tumor syndromes^21^. Specifically, meningiomas with loss of the *NF2* gene show activated mTORC1 signaling as well as an mTORC2-specific serum/glucocorticoid-regulated kinase 1 (SGK1) signaling axis^15–17^. Independent of mTORC1/2 activation, a high-throughput kinome screen conducted on *NF2*-null human arachnoidal and meningioma cells revealed activation of erythropoietin-producing hepatocellular (EPH) receptor tyrosine kinases (RTKs), c-KIT, and SRC family kinase (SFK) members^8,22^. Based on these results, a study administering combination therapy comprising the dual mTORC1/2 inhibitor AZD2014 and dasatinib, a multi-kinase inhibitor targeting SFKs, several EPH receptors and c-Kit^23^, was performed on *NF2*-deficient meningioma cells^22^. The combination of AZD2014 and dasatinib effectively controlled the growth of *NF2*-deficient meningioma *in vitro* and *in vivo*. Two clinical trials of AZD2014 for NF2-associated and sporadic meningioma are currently underway (NCT02831257, NCT03071874).

For these reasons, we investigated the effects of AZD2014 and/or dasatinib in the context of the activated mTORC1/2 and EPH receptor-mediated signaling observed in sporadic and NF2-associated VS. We show that combination therapy is more effective at reducing the metabolic activity of primary human VS cells than either drug alone *in vitro*. In a mouse model of schwannoma, combined treatment with AZD2014 and dasatinib is more effective at inhibiting tumor growth than either monotherapy. Our study demonstrates that co-targeting the mTOR and EPH receptor pathways may constitute a novel therapeutic strategy for VS.

## Materials and Methods

### Human specimen collection and primary cell culture

Surgical vestibular schwannoma (VS) and great auricular nerve (GAN) specimens were obtained from patients undergoing indicated procedures at Massachusetts General Hospital and Massachusetts Eye and Ear. The study was conducted in accordance with the Helsinki Declaration of 1975 and written informed consent was obtained from all subjects prior to inclusion. GAN (control) samples were obtained from patients undergoing benign parotidectomy unrelated to VS, during which this nerve is routinely sacrificed. Patients who had received radiation therapy prior to surgery were excluded. All VS and GAN samples were received and processed according to protocols approved by the Human Studies Committee of Massachusetts General Hospital and Massachusetts Eye and Ear (Board Reference #14–148 H). Following surgical resection, VS or GAN tissue was immediately placed in saline solution, transported to the laboratory, and cultured. Our methods describing precise protocols for surgical specimen collection, processing, and primary cell culture are published in detail^24,25^. Mouse *Nf2*^−/−^ Schwann cells used in our mouse allograft model were maintained in 10% Schwann cell medium containing Schwann cell growth supplement (SCGS, ScienCell).

### Immunoblotting

Protein lysates were prepared from fresh surgical VS and GAN specimens directly after receiving samples from the operating room. Briefly, on ice, total protein was extracted from tumor or nerve using RIPA lysis buffer supplemented with protease and phosphatase inhibitors (Roche Applied Sciences), as previously described^6^. Protein lysates for cultured SCs and mouse tumors were prepared using the same lysis conditions as VS and GAN samples. The resulting lysates were isolated by centrifugation and stored at −80 °C. Protein lysates were resolved by SDS-PAGE as previously described^16^. Commercial antibodies included pEPHA2(S897), c-KIT, pSRC/SFK(Y416), SRC, pAkt(S473), Akt, SGK1, pNDRG1(T346), pS6(S240/244), and S6 (Cell Signaling Technology); NDRG1 (Abcam); EPHA2 (Santa Cruz Biotechnology); GAPDH (EMD Millipore); and β-actin (Sigma Aldrich). NF2/merlin polyclonal C26 antibody has been previously described^26^. Quantitation of immunoblotting was performed using ImageJ/Fiji software^27^.

### CRISPR/Cas9 genome editing of immortalized human schwann cells

**Figure 1:**
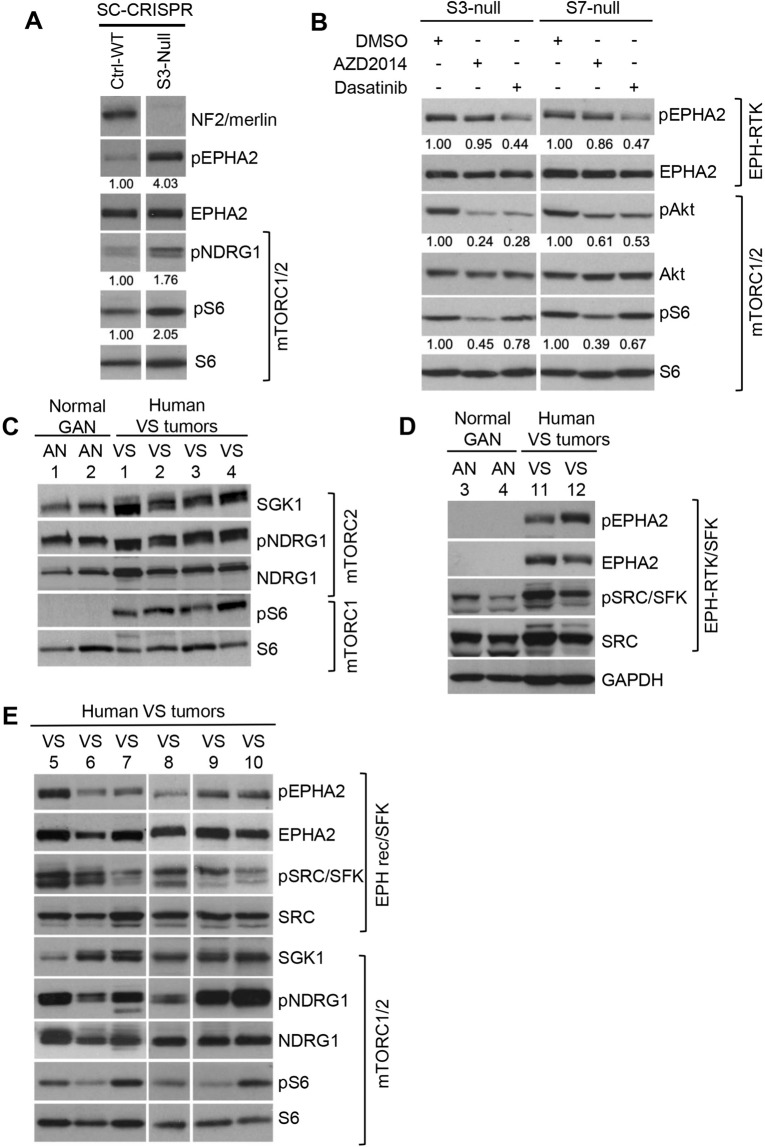
mTOR and EPH receptor signaling is activated in primary human VS and human models of NF2-deficient schwannoma. (**A**) Immunoblotting of human NF2-null SC-CRISPR cells show loss of NF2 and increased pS6^S240/244^ (mTORC1 readout), pNDRG1^T346^ (mTORC2 readout) and pEPHA2^S897^ compared to NF2-expressing control. (**B**) Immunoblotting of two independent SC-CRISPR clones (S3-null and S7-null) treated with AZD2014 (0.3 µM, 24 h) show attenuation of mTORC1/2 readouts pS6 S240/4 and pAkt S473, respectively) compared to DMSO vehicle control. In addition, treatment with dasatinib (0.1 µM, 24 h) demonstrated downregulation of pEPHA2 S897 and pAkt S473). Immunoblot quantitation, performed using ImageJ/Fiji, is shown above the blots (**A**,**B**). (**C**) Four primary human vestibular schwannomas (VS1-4) demonstrate increase in AZD2014 targets mTORC1 (pS6 readout) and mTORC2 (SGK1, pNDRG1 readouts) signaling compared to 2 normal human great auricular nerve samples (AN1-2). (**D**) An additional two primary human VS (VS11-12) demonstrated increased phosphorylation of dasatinib target pSrc/SFK compared to 2 normal human AN (AN3-4). While dasatinib target pEPHA2 along with total EPHA2 were also observed in VS, EPHA2 expression remained below detectable level in AN samples. (**E**) Immunoblotting of 6 additional human VS (VS5-10) tumors revealed variable levels of pEPHA2 and pSrc/SFK^Y416^ along with mTORC1/2 readouts.

To generate isogenic *NF2*-expressing and *NF2*-null Schwann cells (SCs), we utilized an immortalized human SC line, pn02.3 previously described^17,28^. Briefly, lentiviral packaging of the human NF2_sg1 single guide RNA (sgRNA) targeting *NF2* exon 8 cloned into the lenti-CRISPR backbone (a kind gift from the Zhang laboratory at the Broad Institute and MIT) was carried out as described^29^. Lentiviral transduction of human immortalized SCs was carried out by spin-infection followed by puromycin selection as previously described^15^. Single clones were picked, expanded, and genomic DNA was extracted for Sanger sequencing. Sanger sequencing of *NF2* exon 8 in two clones (termed S3-null and S7-null) revealed a homozygous 316 bp deletion (cDNA: 803del316 bp; aa: 268 > fs X) in S3-null and a homozygous 16bp deletion (cDNA: 797del16bp; aa: 266 > fs X) in S7-null, both of which resulted in loss of NF2 protein (see Fig. 1A and Supplementary Fig. S1 for immunoblotting of NF2/merlin).

### Drug preparation and *in vitro* treatment

For *in vitro* studies, primary VS cultures were treated with AZD2014 (provided by AstraZeneca; Wilmington, DE; CAS No. 1009298-59-2) and dasatinib (Selleck Chemicals; CAS No. 302962-49-8). Drugs were dissolved in dimethyl sulfoxide (DMSO) with a final concentration of 0.1% on cells for drug treatment and vehicle controls. See figure legends for final drug concentrations and treatment times on cells. Dose-response experiments were performed on primary cells within two weeks of establishing viable cultures to ensure maximal schwannoma cell purity^24^.

### *In vitro* cytotoxicity and cell confluence assays

Following drug treatment, *in vitro* toxicity of primary VS cells was assessed using the colorimetric 3- (4,5-dimethylthiazol-2-yl) -2,5-diphenyltetrazolium bromide (MTT) assay (Life Technologies), according to the manufacturer’s instructions. All drug treatments were assessed in 3–5 technical replicates per drug concentration per tumor. The optical density (OD) of each well was read at 570 nm using a spectrophotometer. The OD values of wells exposed to vehicle (0.1% DMSO) were averaged and set to 100% and used to normalize OD values of cells treated with drugs; metabolic activity was then reported as percent change from control. In accordance with good statistical practice in pharmacology, all statistical testing was performed on raw OD values (see *Statistical Analysis*).

Cell confluence was measured using live-cell, time-lapse phase contrast imaging acquired at 10X by an IncuCyte S3 instrument (Essen Bioscience). Nine images were acquired per well per tumor every 2 hours for the duration of the 72-hour drug treatment period. Phase object confluence, a measurement of cell confluence per square millimeter of well space, was then calculated using IncuCyte software (Essen Bioscience) and reported.

To test for a possible synergistic effect between AZD2014 and dasatinib in primary VS cells and S3 *NF2*-null cell lines we used the widely accepted Chou-Talalay’s combination index (CI) method for quantifying synergistic or antagonistic drug interactions, implemented in CompuSyn freeware^30^. This method is based on the median-effect equation and the combination index equation, which together define fractional inhibition of metabolic activity (known as “fraction affected”, Fa), drug interaction depending on the CI value (synergistic: CI < 1, additive: CI = 1, antagonistic: CI > 1) and dose-reduction index, DRI^31^.

### Animal models and treatment protocol

All animal procedures were performed following the guidelines of Public Health Service Policy on Humane Care of Laboratory Animals and approved by the Institutional Animal Care and Use Committee of the Massachusetts General Hospital. To reproduce the microenvironment of peripheral schwannomas, we implanted *Nf2*^−/−^ mouse Schwann cells into the sciatic nerve of syngeneic immunocompetent FVB/C57BL/6 mice at 10–12 weeks old^12,32^. Both male and female mice were used in this study. All mice were bred and maintained at the Gnotobiotic Mouse Cox 7 Core at Massachusetts General Hospital (https://researchcores.partners.org/cox/about). Mouse *Nf2*^−/−^ Schwann cell suspension (5 × 10^4^ cells in 3 μL) was injected slowly (over 45–60 seconds) under the sciatic nerve sheath using a Hamilton syringe to prevent leakage. AZD2014 (15 mg/kg, diluted in 1% Tween 80 at the concentration of 5 mg/ml) and/or dasatinib (15 mg/kg diluted in 80 mM citric acid, at a concentration of 5 mg/ml) was administered by oral gavage every day and continued until study endpoint. Tumor size was measured by caliper every 3 days until tumors reached 1 cm in diameter. Tumor growth delay is a widely used method for assessment of tumor treatment modalities as it permits a quantitative evaluation of treatment-induced alterations of tumor-growth patterns^33–35^. Tumor growth delay was calculated for treatment groups relative to control tumors, and measured in number of days required for a tumor to grow to 1 cm in diameter.

### Statistical analysis

Though metabolic activity and cellular proliferation data are presented as percentage of vehicle-treated control, all statistical analyses were performed on raw data, in accordance with good statistical practice in pharmacology. Significant differences in tumor growth between two groups were analyzed using the Student’s *t* test (two-tailed) or Mann-Whitney *U* test (two-tailed) with p < 0.05 considered significant.

## Results

### mTOR and EPH receptor signaling is activated in primary human VS and human models of NF2-deficient schwannoma

We have previously shown that, similar to NF2 loss in AC and MN cells, NF2-suppression using short hairpin RNA (shRNA) in SCs also leads to constitutive activation of mTORC1 and mTORC2-SGK1 signaling^15–17^. Expanding on those studies, we carried out CRISPR/Cas genome editing in immortalized human SCs to generate isogenic SC-CRISPR cells, *NF2-*expressing and *NF2*-null. Consistent with our previous results, *NF2*-null SCs showed loss of NF2 protein expression as well as a marked increase in mTORC1 signaling (evidenced by upregulation of pS6)*,* mTORC2 signaling (evidenced by upregulation of pNDRG1 that is phosphorylated by SGK1, a direct target of mTORC2) and phosphorylated EPH receptor tyrosine kinase (RTK) EPHA2 (pEPHA2) compared to *NF2*-expressing CRISPR control (Ctrl-WT) (Fig. 1A and Supplementary Fig. S2). Treatment of two independent *NF2*-null SC-CRISPR clones, S3-null and S7-null, using the dual mTORC1/2 inhibitor AZD2014 demonstrated attenuation of pS6 (mTORC1 readout) and pAkt (another mTORC2 readout), but had no effect on EPH-RTK pEPHA2. Conversely, dasatinib treatment led to downregulation of pEPHA2 and pAkt, a known downstream effector of EPH-RTK signaling, but showed minimal effect on mTORC1 signaling (Fig. 1B and Supplementary Fig. S1). Next, we examined mTORC1/2, EPH-RTK and pSrc/SFK signaling in primary human VS tumor (VS1-12, Table S1) samples compared to great auricular nerve (GAN). As a peripheral, sensory nerve, schwannomas of the GAN are exceptionally rare and GAN constitutes a maximally relevant control nerve against which to compare VS gene and protein expression^25^. We observed that NF2-associated primary VS tumors demonstrated robust activation of pS6 (mTORC1) compared with GANs, which were negative for pS6. In addition, VS tumors showed robust expression of SGK1, a direct phospho-target of mTORC2, along with pNDRG1, the downstream target of SGK1, suggesting that mTORC1/mTORC2 signaling plays a significant role in VS pathobiology (Fig. 1C and Supplementary Fig. S2). Immunoblotting of pSrc/SFK demonstrated an increase in primary VS tumors compared to GAN samples, and we also observed pEPHA2 in primary VS tumors, however expression of the EPHA2 receptor was not detectable in GAN samples by immunoblotting (Fig. 1D and Supplementary Fig. S3). Immunoblotting of an additional 6 primary VS tumors demonstrated variable levels of mTORC1/2 signaling readouts pS6, SGK1, and pNDRG1, as well as pEPHA2 and pSrc/SFK (Fig. 1E and Supplementary Fig. S4). Taken together, we have observed expression and activation of mTORC1/mTORC2, EPHA2 and Src/SFK in all human VS tumors tested, suggesting a therapeutic role for targeting these pathways.

### Combination AZD2014 and dasatinib therapy synergistically reduces the metabolic activity of primary human VS cells

**Figure 2:**
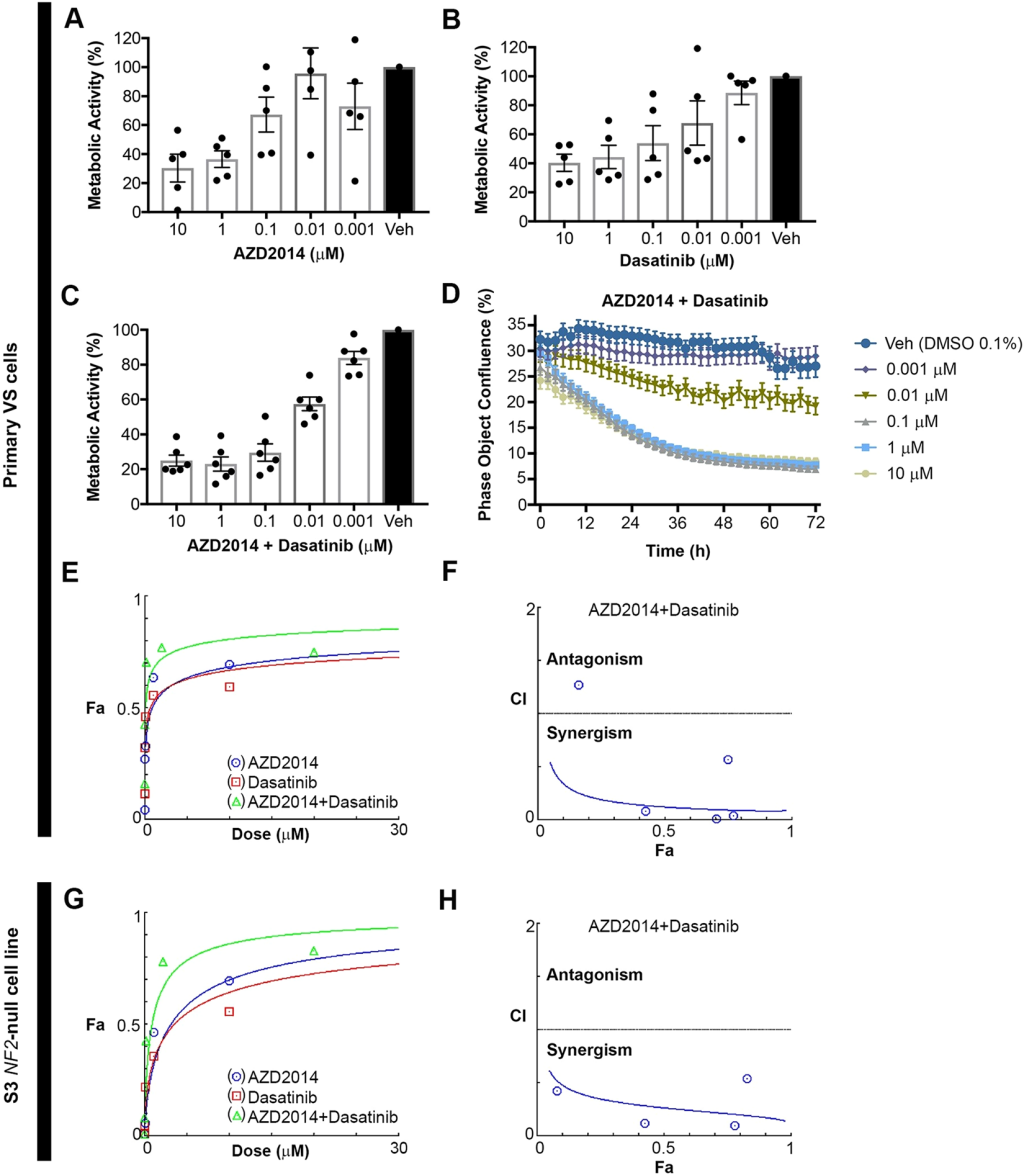
AZD2014 and dasatinib therapy in combination reduces the metabolic activity of primary human VS cells more consistently and significantly than either drug alone. (**A**) Metabolic activity of primary VS cells cultured from 5 human tumors treated for 72 h with increasing concentrations of AZD2014 alone (measured via MTT assay). (**B**) Metabolic activity of primary VS cells cultured from 5 human tumors treated for 72 h with increasing concentrations of dasatinib. (**C**) Metabolic activity of primary human cells cultured from 6 human tumors treated for 72 h with increasing concentrations of AZD2014 and dasatinib together. Treatment with combination therapy collapses the variance apparent when treating tumor cells with either drug alone. (**D**) Live-cell imaging of primary VS cells treated with combination AZD2014 and dasatinib therapy every 2 hours for 72 hours shows a decrease in phase object confluence over time proportional to the expected dose-response. Veh, vehicle control (0.1% DMSO). All drugs were diluted to the concentration of interest in VS cell growth medium (DMEM/F12, 10% fetal bovine serum, 1% penicillin/streptomycin mix) and applied to primary VS cells (24-well plate, 1 mL drug-containing medium per well) for 72 hours. (**E**) Dose-response curves for primary VS cells, calculated using CompuSyn software (Chou-Talalay method), shows that the fractional inhibition of metabolic activity, Fa, is substantially higher for AZD2014 and dasatinib equimolar drug combination (depicted in **C**) than for either drug alone. (**F**) Combination index (CI) plots for AZD2014 and dasatinib equimolar drug combination demonstrates strong synergism (CI < 1) for all but the lowest drug concentration where weak antagonism is observed (CI > 1). Horizontal line at CI = 1 indicates an additive effect. (**G**) Dose-response curves for S3 NF2-null SC-CRISPR cells treated with five different equimolar concentration of AZD2014 and dasatinib ranging from 0.002–10 µM for 72 h. Fa is substantially higher for AZD2014 and dasatinib equimolar drug combination than for either drug alone. (**H**) Combination index plot for AZD2014 and dasatinib equimolar drug combination applied to S3 cells demonstrates clear synergism across all tested drug concentrations.

After establishing that mTORC1/2 signaling and EPH receptor/SFK signaling is consistently observed in human VS, we treated primary human VS cells (VS13-28, Table S1) grown from surgical tumor samples with dual mTORC1/2 inhibitor AZD2014 and the tyrosine kinase inhibitor dasatinib. We assessed the metabolic activity of five independent primary VS cultures after treatment with AZD2014 alone (Fig. 2A), five independent VS cultures after treatment with dasatinib alone (Fig. 2B), and six independent VS cultures after treatment with equimolar concentrations of AZD2014 and dasatinib administered in combination (Fig. 2C,D). Patient demographic information, hearing status, and MRI scans are comprehensively included as Supplementary Material (Supplementary Tables S1, S5).

We independently validated the dose-response results observed in primary human VS cells treated with combination AZD2014 and dasatinib therapy (Fig. 2C) by using live-cell imaging to capture a similar dose-dependent decrease in cell confluence over time (Fig. 2D). Sequential phase-contrast live-cell imaging was performed every 2 hours during a 72-hour treatment period, and the percentage of the area of each well covered by cells at the time of each scan was reported as phase object confluence. Figure 2D displays representative data from one tumor, in which live-cell imaging results align closely with the observed dose-dependent decrease in metabolic activity. In primary VS cells treated for 72 h with vehicle alone, we observed a stable cellular confluence, presumably due to the slow growing nature of these cells in the assay time period. Treatment with 0.001 µM of combination therapy appeared nearly indistinguishable from vehicle-treated cells, while VS cells treated with 0.01 µM demonstrated approximately half of the maximum effect. Increasing the drug concentration above 0.1 µM did not seem to increase the efficacy of combination therapy. Estimated IC50 concentration when both AZD2014 and dasatinib were applied together in equimolar concentration (IC50 = 0.09 μM) was dramatically reduced compared to IC50 for AZD2014 alone (0.87 μM) or dasatinib alone (0.62 μM), consistent with synergistic drug interaction (Fig. 2E). Very strong synergism was observed for equimolar drug concentrations ranging from 0.01 µM to 1 µM (CI: 0.013–0.082, Fa: 0.42–0.77), which reduced metabolic activity more than 40% (Fig. 2F, Table 1). The synergistic effect of the drugs was followed by the favorable dose reduction (DRI > 1) at all tested concentrations except for dasatinib at the lowest applied concentration (DRI < 1) (Table 1). The highest dose reduction for both drugs was observed when they were applied at 0.1 µM equimolar concentration in primary VS cells, causing 70% inhibition of their metabolic activity. This effect is achieved by 135.2 fold and 192.7 fold lower concentrations of AZD2014 and dasatinib, respectively than if they were applied separately.

**Table 1:** Synergy parameters of AZD2014 and Dasatinib applied together at equimolar concentrations to primary VS cells and S3 *NF2*-null SC-CRISPR cells.

Cells	AZD2014+ Dasatinib (µM)	Fractional inhibition (Fa)	CI	Description	DRI (AZD2014)	DRI (Dasatinib)
Primary VS cells	0.001 + 0.001	0.161	1.272	Moderate antagonism	4.798	0.941
	0.01 + 0.01	0.425	0.082	Very strong synergism	33.542	18.990
	0.1 + 0.1	0.705	0.013	Very strong synergism	135.233	192.714
	1 + 1	0.770	0.039	Very strong synergism	39.297	73.091
	10 + 10	0.750	0.565	Synergism	2.803	4.793
S3 NF2-null SC-CRISPR cells	0.002 + 0.002	0.010*	2.385	Antagonism	2.551	0.502
	0.014 + 0.014	0.082	0.424	Synergism	7.584	3.421
	0.123 + 0.123	0.425	0.120	Strong Synergism	16.518	16.698
	1.111 + 1.111	0.780	0.095	Very strong synergism	16.301	29.971
	10 + 10	0.828	0.537	Synergism	2.765	5.707

Combination index (CI) and dose- reduction index (DRI) were calculated from CI- and DRI- equation algorithms using CompuSyn software (Chou-Talalay CI method)^30^. CI < 1, C = 1, and C > 1 indicate synergism, additive effect, and antagonism, respectively. DRI < 1, DRI = 1, and DRI > 1 indicate not favorable dose-reduction, no dose-reduction, and favorable dose-reduction, respectively. Fa- fraction affected (i.e. fractional inhibition of metabolic activity). *This is an approximation because the actual value was below 0.01, which is the lowest value allowed by the algorithm.

We next used the immortalized *NF2*-null SC-CRISPR cell line S3-null to additionally validate the synergy between the AZD2014 and dasatinib drug effect. A major advantage of an immortalized cell line is that much larger numbers of cells can be utilized than available from a primary cells derived from a typical VS tissue sample. Consequently, in addition to performing dose response experiments with select drug concentrations (Fig. 2G,H), a full dose-response matrix can be tested to precisely define synergy scores (Supplementary Fig. 6). When using the appropriate drug concentrations range as utilized in the primary VS cells, the S3 cell line was markedly less sensitive to AZD2014 alone (IC50 = 3.09 μM), dasatinib alone (IC50 = 3.51 μM) and equimolar concentration of AZD2014 and dasatinib (IC50 = 0.87 μM) than primary VS cells (Fig. 2G). Nonetheless, the synergistic effect was present in the S3 cell line, was very strong at 1.11 µM drug concentration (CI:0.095, Fa: 0.78) (Fig. 2H, Table 1B), and was accompanied by the favorable drug dose reduction (DRI > 1).

Next, we quantified the degree of drug synergy in S3 cell line by calculating synergy scores using a 6 × 6 dose–response matrix resulting in 36 conditions (Supplementary Fig. 6) because 6 different drug concentrations were used in the previous synergy testing. Synergy scores were calculated as deviation of the observed metabolic inhibition caused by drug treatment and expected inhibition under drug non-interaction assumptions determined by four different synergy scoring models including Highest Single Agent (HAS), Loewe Additivity, Bliss Independence and Zero Interaction Potency (ZIP) using the SynergyFinder web application^36^. Malyutina, *et al*. classifies drug combinations that show synergy score higher than 5 or lower than −5 in all four models as true synergism or antagonism, respectively^37^. For S3 null cells, synergy scores calculated by all four reference models were 5 9.5 (HSA), 31.8 (Loewe), 5.2 (Bliss) and 5.5 (ZIP) (Supplementary Fig. 6C) indicating that AZD2014 and dasatinib synergistically inhibit metabolic activity of VS cells.

### Combined AZD2014 and dasatinib treatment significantly inhibits tumor growth in a mouse allograft schwannoma model

**Figure 3:**
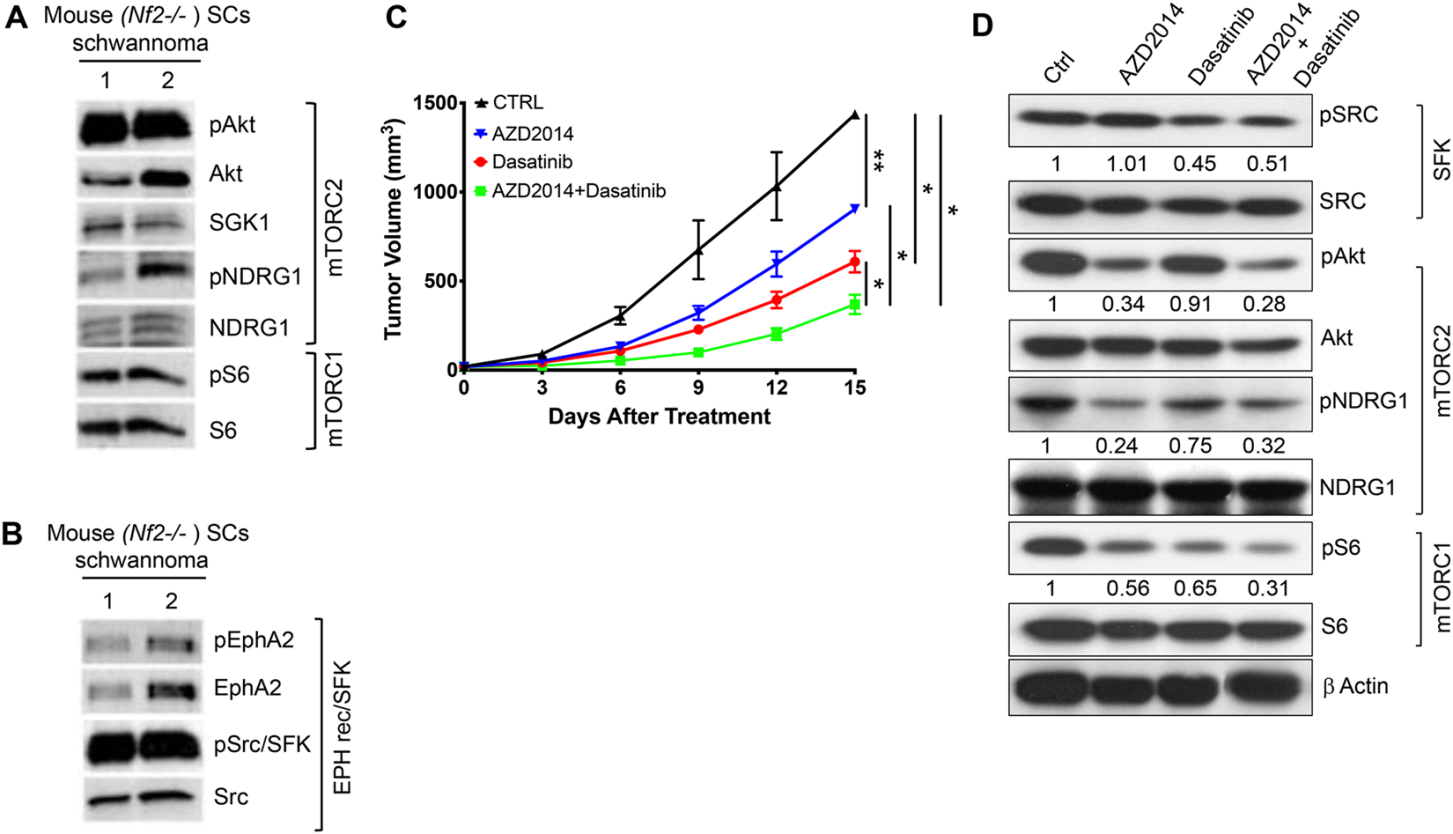
Combined AZD2014 and dasatinib treatment significantly inhibits tumor growth in a mouse allograft schwannoma model. (**A**) Immunoblotting of *in vivo* mouse schwannomas from 2 independent Nf2^−/−^ Schwann cell (SC)-implanted tumors show activated mTORC1 (pS6) and mTORC2 (pAkt^S473^, SGK1, pNDRG1) signatures. (**B**) Immunoblotting of mouse allograft schwannomas also show activated pEPHA2 and robustly activated pSrc/SFK. (**C**) Tumor growth delay, defined by the time required for tumors to reach 1 cm in diameter. Vehicle-treated mice (n = 13); mice treated with AZD2014 alone (15 mg/kg, n = 16); mice treated with dasatinib alone (15 mg/kg, n = 16); and mice treated with a combination of AZD2014 and dasatinib (n = 14). Representative data from two independent experiments; mean ± SEM. (**D**) Immunoblot of *in vivo* mouse schwannomas from different treatment groups confirmed that targets of AZD2014 and dasatinib were inhibited. Immunoblot quantitation, performed using ImageJ/Fiji, is shown above the blots.

To evaluate the efficacy of AZD2014 and dasatinib *in vivo*, we first confirmed the mTORC1/2 signaling pathways readouts in our *Nf2*^−/−^ mouse allograft schwannoma model. Immunoblot data from mouse tumors demonstrated activated signatures of mTORC1 and mTORC2 pathway members (Fig. 3A), matching our results in *NF2*-null SCs (Fig. 1A,B) and human VS tumor samples (Fig. 1C,E). Similarly, mouse tumors also demonstrated activated pEPHA2 and robustly activated pSrc/SFK **(**Fig. 3B), consistent with the pattern observed in human VSs (Fig. 1D,E). These data suggest that our model successfully recapitulates the drug-relevant mTORC1/2 and EPH-RTK/SFK pathways observed in our human VS tumor samples.

Next, we compared the efficacy of AZD2014 and dasatinib monotherapies with that of combination therapy (AZD2014 and dasatinib administered together). In our *Nf2*^−/−^ mouse allograft schwannoma model, none of the monotherapies or combination treatment stopped tumor growth (Supplementary Fig. S7). However, AZD2014 and dasatinib monotherapies each induced significant tumor growth delay. Combination therapy with AZD2014 and dasatinib resulted in the most dramatic tumor growth delay (p < 0.001), a response significantly more effective than that produced by either monotherapy **(**Fig. 3C). Immunoblot analysis of the tumor from different treatment groups confirmed that targets of AZD2014 and dasatinib were inhibited *in vivo* (Fig. 3D).

## Discussion

Identification of a safe, effective, non-invasive drug therapy to treat sporadic and NF2-associated VS would prove transformative for patients with very limited options. Further, the identification of a therapeutic regimen that could potentially inhibit the growth of multiple tumor types associated with NF2 (VS and meningioma) constitutes an urgent unmet need. We provide evidence that AZD2014 and dasatinib, when administered together, can potently diminish the metabolic activity of primary human vestibular schwannoma cells and delay the growth of *NF2*-null tumors in a mouse allograft model of schwannoma, and that combination therapy in all cases is more effective than either drug alone. Moreover, this drug combination exhibits a therapeutically promising effect on VS cells at a physiologically reasonable concentration (~0.1 µM), a concentration effectively identical to that used to control the growth of *NF2*-null meningioma.

Earlier studies have shown activation of Src in NF2 wild-type vs NF2 deficient Schwann cell lines^38^ and treatment of merlin-deficient mouse Schwann cell lines also identified dasatinib as a potentially effective agent based on the results of a high-throughput drug screen^39^ however, this study focused on a more specific Src inhibitor, saracatinib, in combination with the c-Met inhibitor cabozantinib^39^. Dasatinib is FDA-approved for treatment of acute lymphocytic leukemia and chronic myeloid leukemia with mutant Abl kinase expression, and is shown to inhibit oncogenic and invasive processes in blood cancers and solid tumors^40^. Dasatinib was first characterized as a potent Src/Abl inhibitor, and subsequently has also been shown to target receptor tyrosine kinases (RTKs) including PDGFR, KIT and EPH receptor family members EPHA2 and EPHB1^23^. PI3K-Akt signaling is activated by RTKs, such as PDGFR and KIT, as well as non-receptor Src family kinases (SFKs)^41,42^, and therefore by inhibiting these upstream kinases, dasatinib treatment can lead to attenuation of activated Akt. Our study confirmed expression of AZD2014 and dasatinib targets in all human VS tumors tested, and we therefore chose to further evaluate dasatinib because it has the advantage of simultaneously inhibiting multiple signaling pathways observed in NF2-associated tumors. Moreover, co-targeting these pathways may show superior efficacy to overcome potential compensatory response compared to monotherapeutic strategies.

To test the *in vivo* efficacy, we used mouse *Nf2*^−/−^ Schwann cells isolated from *Nf2*loxP/loxP mice at embryonic day 13.5^43^. Current available schwannoma cell lines also include HEI-193 and SC4 cells. HEI-193 cell was established at House Research Institute by David Lim’s team^44^ and SC4 cell was developed by Marco Giovannini’s team. Although the three cell lines are well characterized and used in many publications^45^, all of them have adapted a malignant, aggressive growth behavior, and do not represent the benign schwannoma. While appropriate animal models for NF2 have proved challenging, to better mimic clinical phenotype and accelerate clinical translation, future studies would benefit from use of genetically modified mouse models^45–50^ or establishment of patient-derived xenograft models.

To evaluate the efficacy of combined AZD2014 and dasatinib *in vivo*, we used the sciatic nerve model, which is a widely used animal model in NF2 research^51^. The sciatic nerve model reproduces the microenvironment of peripheral schwannomas and the implantation procedure is technically straightforward. However, the sciatic nerve model has several important drawbacks. First, it does not reflect the symptoms induced by vestibular schwannomas (including hearing loss and dizziness). Second, using this model, evaluation of the neurological function is limited to rotarod assay, which only assesses motor coordination and does not reflect sensory function. Future preclinical efficacy studies using the orthotopic models^52–54^ and NF2 genetically engineered mouse models^45–50^ will complement the current study and facilitate translation to clinic. Though dasatinib has been associated with some toxicity in the clinic, we did not observe systemic toxicity (body weight loss) in mice treated with AZD2014, dasatinib, or combination therapy. Additionally, dasatinib has been shown to have poor nerve penetrance^39^ which provides support for the safety of dasatinib use by NF2 patients. In our model, sciatic nerve was injured during tumor cell implantation, preventing us from accurately evaluating any potential neurotoxicity from AZD2014, dasatinib or the combination treatment. Further research using the genetic engineered models^45–50^ is needed to fully evaluate the neurological effects of treatments.

Targeting the mTOR and EPH/SFK signaling pathways in VS with small molecule drugs also has high potential to be effective when combined with existing therapies, such as radiotherapy or treatment with bevacizumab, a monoclonal antibody targeting vascular endothelial growth factor A (VEGF-A). Bevacizumab treatment causes tumors to shrink in approximately 50% of VS patients; however, this response is often transient and many patients must discontinue treatment due to harmful adverse effects, such as renal failure^55^. Further experiments are necessary to evaluate the effect of AZD2014 and dasatinib therapy when combined with bevacizumab and/or radiotherapy.

Building on our previous findings in *NF2*-deficient meningioma cellular models^22^, our results here in NF2-related vestibular schwannoma further support a strategy to co-target the mTORC1/2 and EPH/SFK signaling pathways and constitutes a compelling new therapeutic avenue for treatment of NF2.

## Supplementary information

Supplementary Material.

## Data Availability

De-identified data generated and/or analyzed during the current study are available from the corresponding author on reasonable request.
