# Supplementary material for: Combination therapy with mTOR kinase inhibitor and dasatinib as a novel therapeutic strategy for vestibular schwannoma
Source: Sci Rep. 2020 Mar 6;10:4211. doi: 10.1038/s41598-020-60156-6 (PMC7060236; doi:10.1038/s41598-020-60156-6)
Supplement: Supplementary file 1 — Supplementary Material. [file 41598_2020_60156_MOESM1_ESM.pdf]

Supplementary Material

Combination therapy with mTOR kinase inhibitor and dasatinib as a novel therapeutic strategy for vestibular schwannoma

Jessica E. Sagers, Roberta L. Beauchamp, Yanling Zhang, Sasa Vasilijic, Limeng Wu, Patrick DeSouza, Richard Seist, Wenjianlong Zhou, Lei Xu, Vijaya Ramesh, Konstantina M. Stankovic

Supplementary Methods

Combination Drug Synergy Testing

In vitro assays were performed to test synergy of AZD2014 and dasatinib in SC-CRISPR S3-null clone as previously described[1]. Briefly, cells were seeded into 384-well plates (400 cells/well) in triplicate. Cells were then treated 24h after seeding with AZD2014 and dasatinib in a 6x6 dose-matrix format using 6 dosage points (0-10µM, 9-fold dilution series). Cell viability assays were performed at 72h post-treatment time using the CellTiter-Glo viability kit (Promega) according to manufacturer's instructions. Luminescence was detected using an EnVision 2103 Multilabel Reader (Perkin Elmer). Single drug dose response curves, combination indexes and synergy scores were calculated using CompuSyn software[2] and SynergyFinder web-based application for analyzing drug combination dose–response matrix data (https://synergyfinder.fimm.fi)[3].

Supplementary Table and Figures

VS	Sex	Age	PTA (ipsi)	WR % (ipsi)	PTA (contra)	WR % (contra)	Longest Lin Dim (mm)
*1	F	29	32	>90	32	>90	30
2	F	44	35	86	35	86	25
3	F	74	5	>92	8	98	29
4	F	51	0	>92	22	92	19
*5	F	18	NT (deaf)	NT (deaf)	2	98	29
6	F	60	NA	NA	NA	NA	NA
7	M	60	45	54	20	92	22
8	NA	NA	NA	NA	NA	NA	NA
9	NA	NA	NA	NA	NA	NA	NA
10	NA	NA	NA	NA	NA	NA	NA
11	M	24	NA	NA	NL	NL	23
12	F	L	35	72	5	96	16
13	M	50	35	22	10	100	25
14	M	40	50	18	13	94	40
15	M	29	40	48	20	98	23
16	F	70	>100 (deaf)	NT (deaf)	10	96	29
17	F	46	80	NT (deaf)	15	100	10
18	M	70	30	62	15	88	37
19	M	52	78	0	17	95	12
20	F	54	8	96	5	98	24
21	M	54	23	92	3	98	11
22	F	52	65	48	22	100	22
23	F	48	28	50	15	92	30
24	M	61	33	88	15	98	10
25	M	81	NT (deaf)	NT (deaf)	NT (deaf)	NT (deaf)	33
26	F	70	18	98	5	100	15
27	F	47	30	60	10	96	15
*28	F	21	95	NT (deaf)	NT (deaf)	NT (deaf)	34

Supplementary Table 1. Patient demographic and tumor-relevant clinical information. VS, vestibular schwannoma (numbering consistent with main text figures); *, neurofibromatosis type 2 (NF2) patient; PTA, pure tone average; WR, word recognition score, measured as percent correct; ipsi, measured from ear ipsilateral to VS; contra, measured from ear contralateral to VS; longest lin dim, longest linear dimension of tumor, measured in mm; NT, not tested; NL, normal; NA, no clinical data made available to researchers.

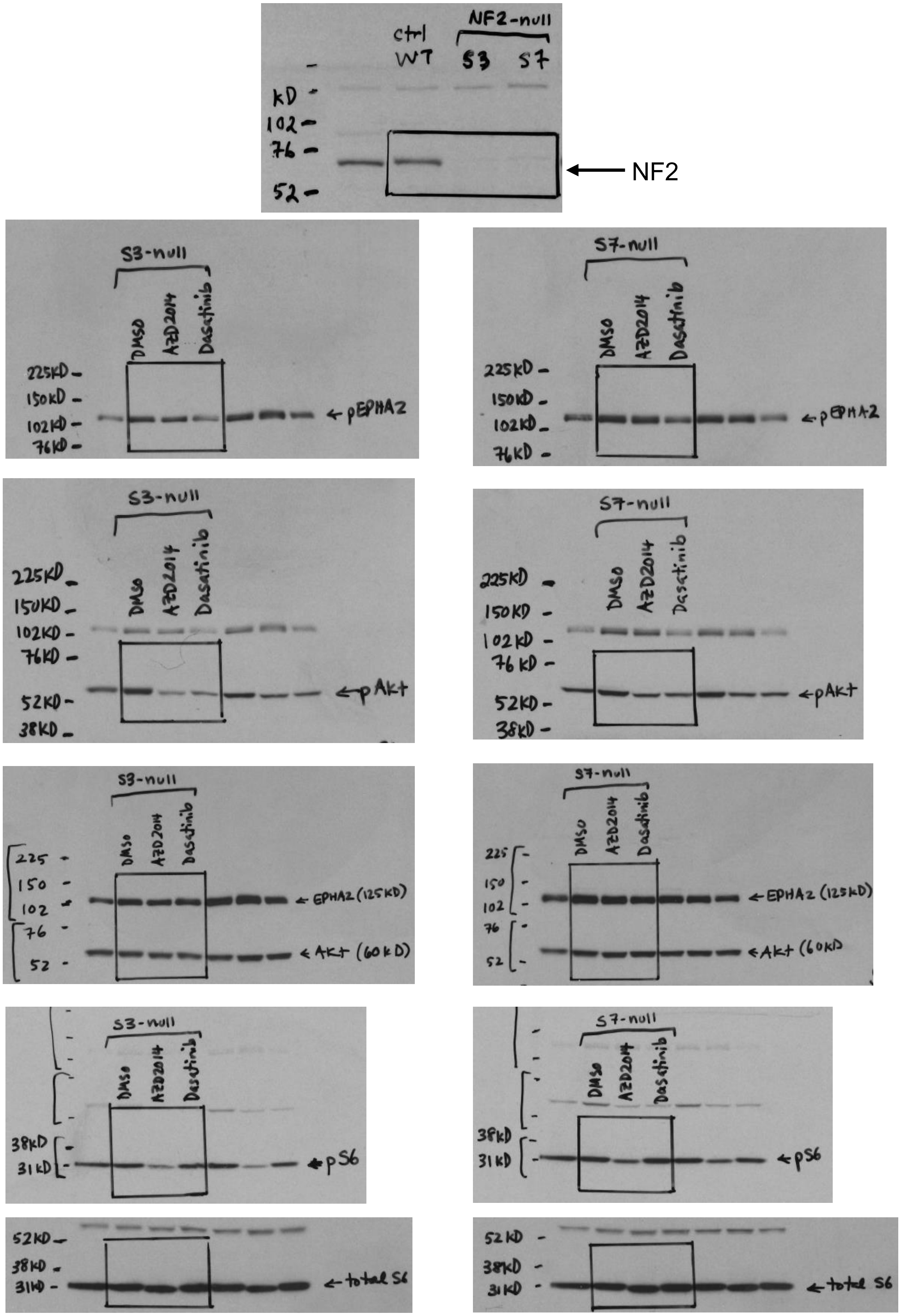

Supplemental Figure S1. Original scans associated with Figure 1B

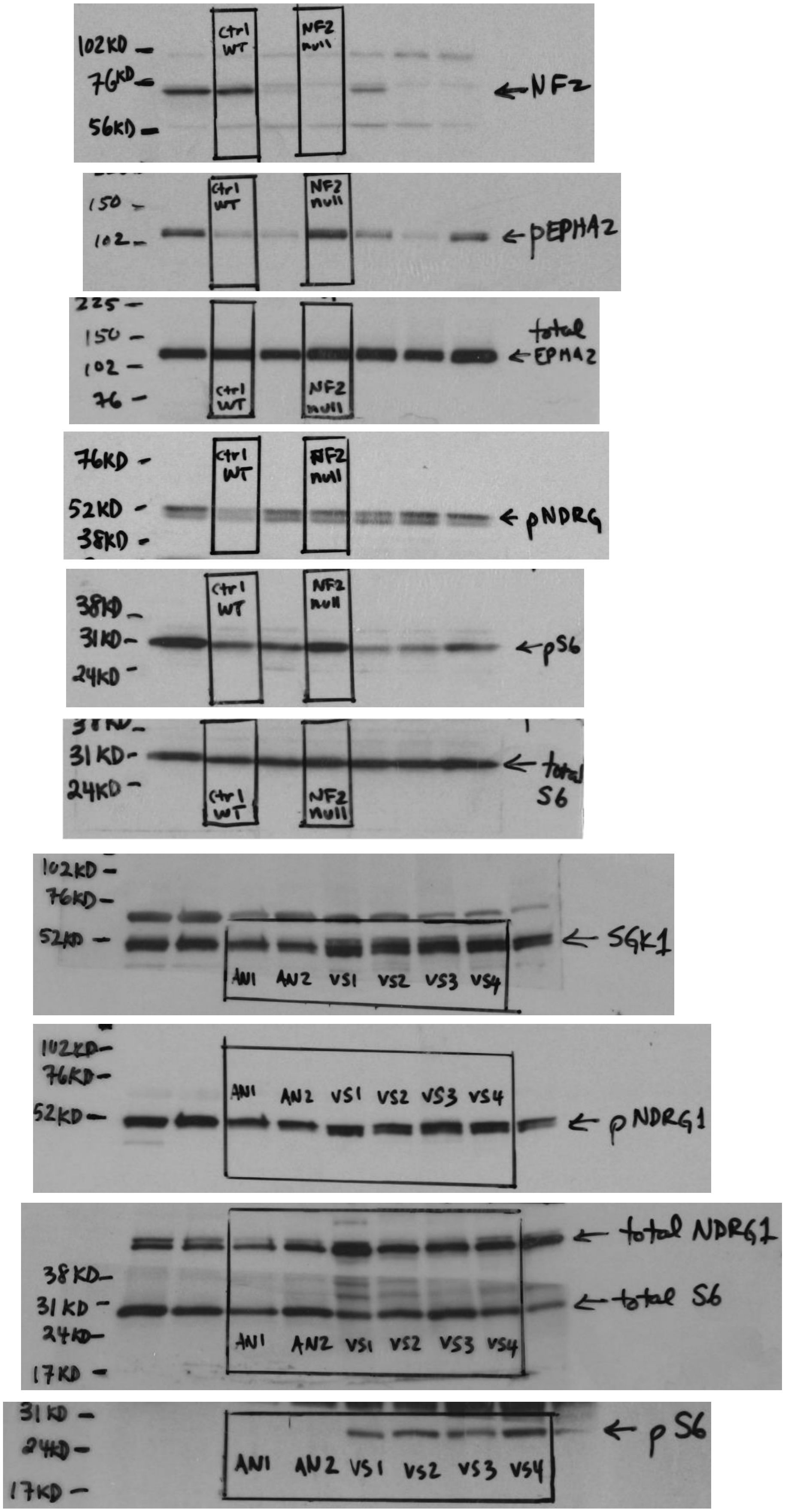

Supplemental Figure S2. Original scans associated with Figure 1A and C

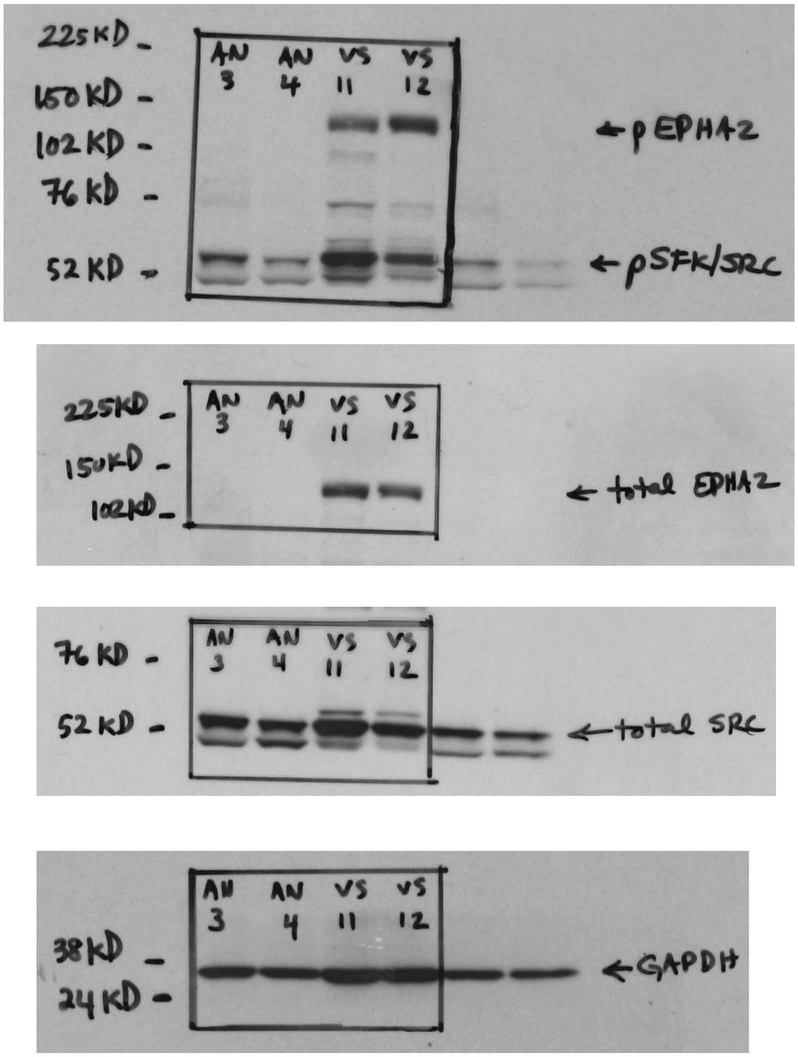

Supplementary Figure S3. Original scans associated with Figure 1D.

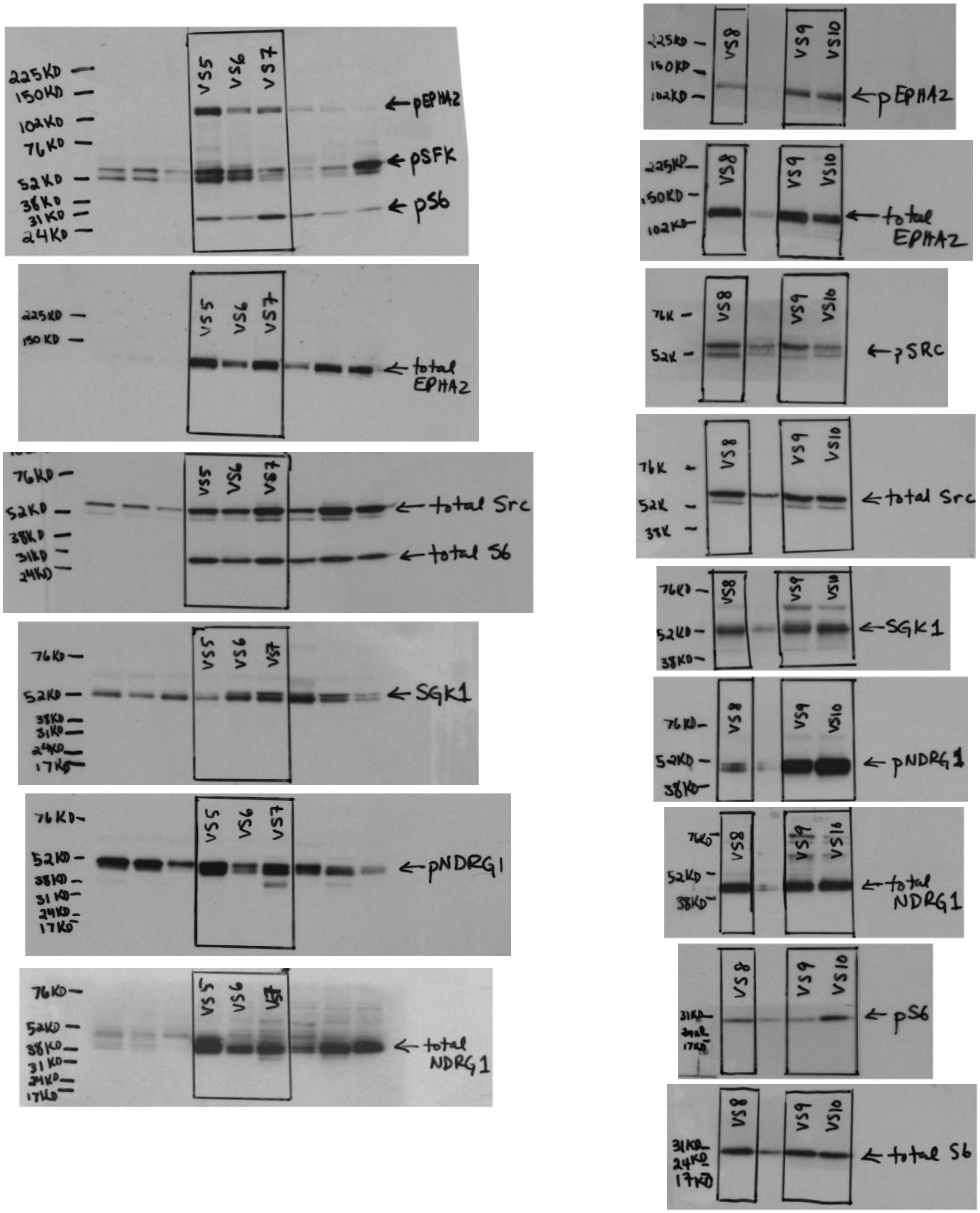

Supplementary Figure S4. Original scans associated with Figure 1E.

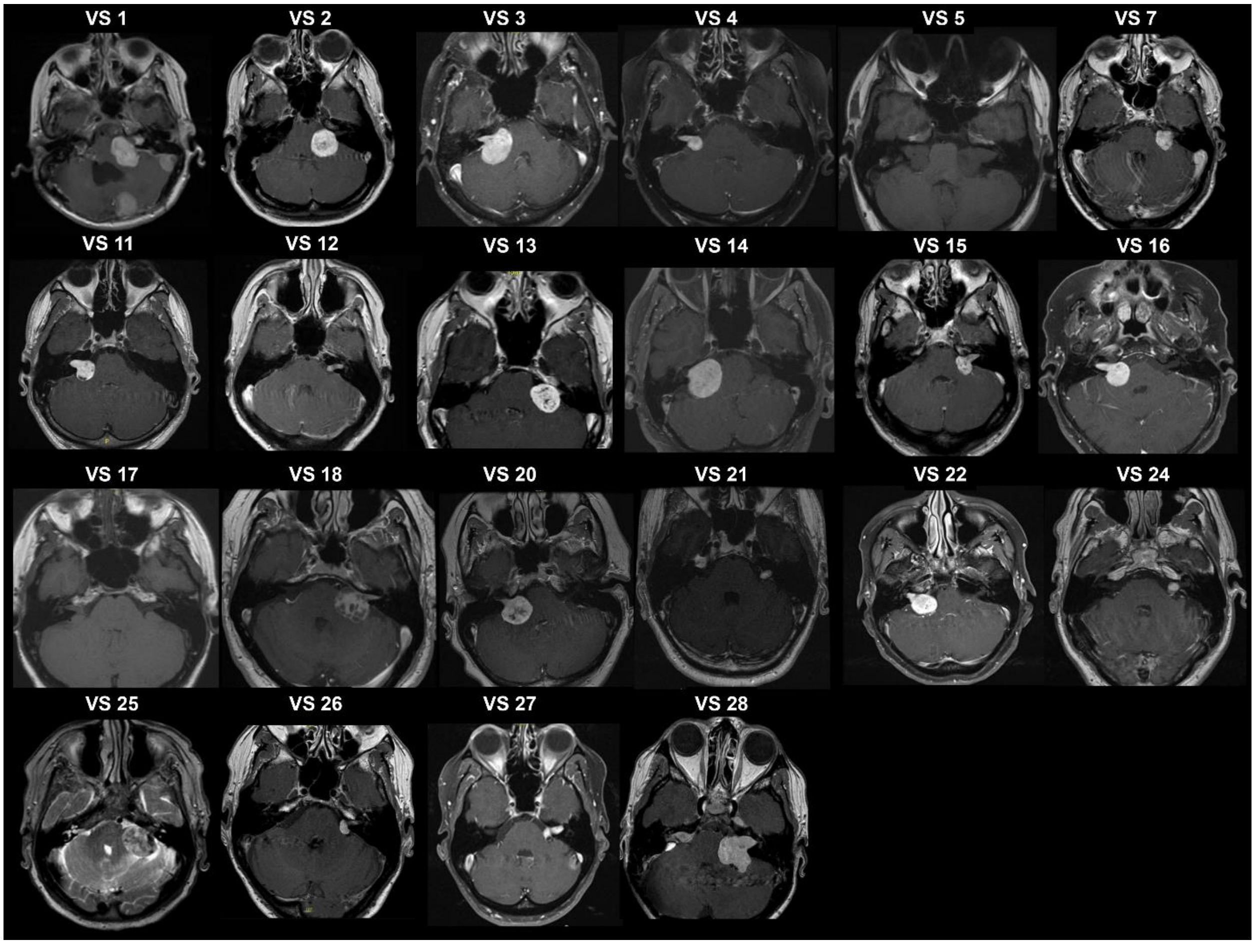

Supplementary Figure S5. Available MRI scans from 22 of 28 VS patients with tumors used in this study. Numbers correspond to those provided in main text figures and to clinical information presented in Supplementary Table 1. T1 weighted scans with contrast are provided wherever available. Tumors avidly take up contrast and appear bright. Scans for VS 5 and VS 17 were performed without contrast dye due to patient intolerance.

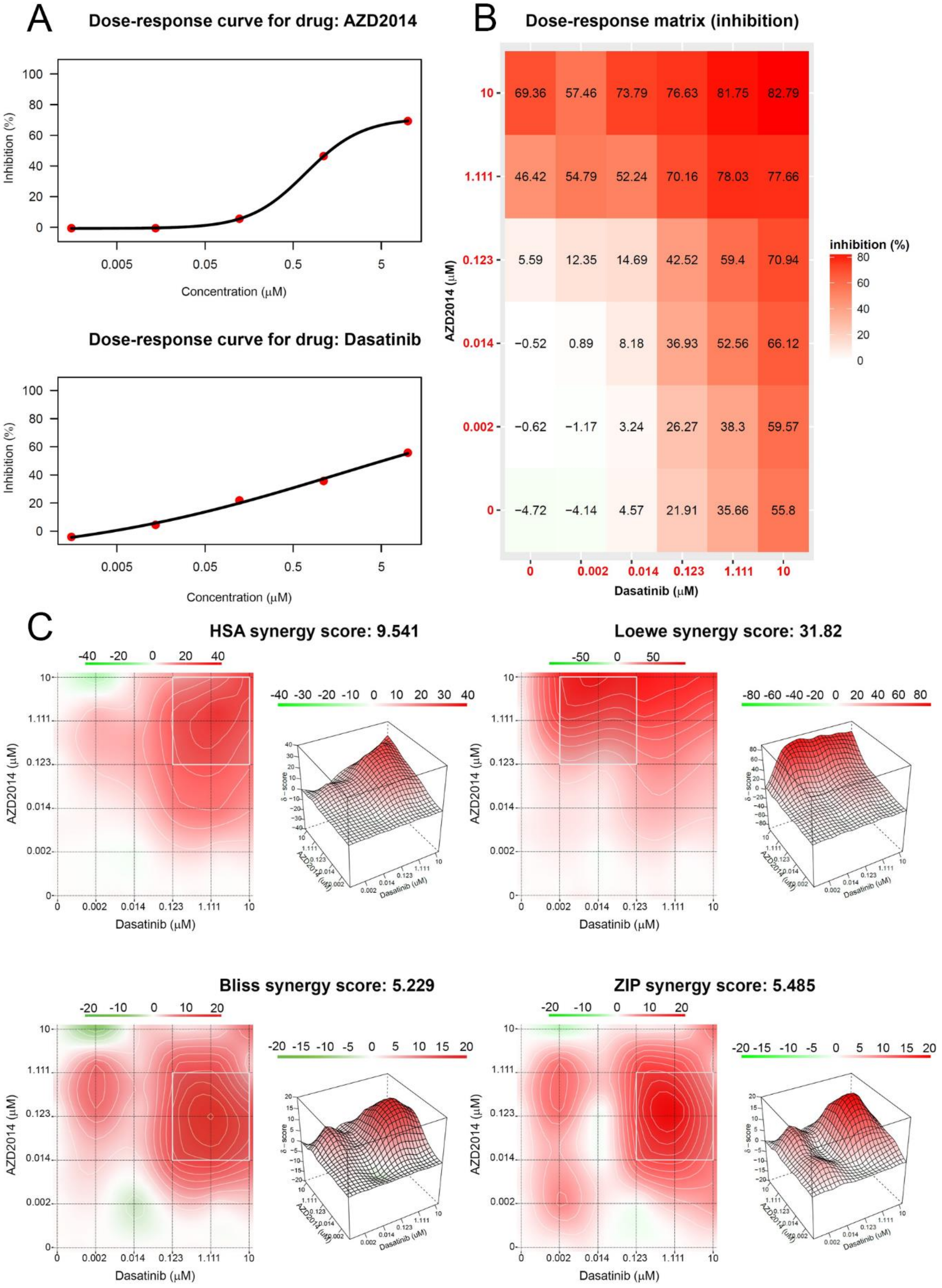
A
Dose-response curve for drug: AZD2014
Inhibition (%)
Concentration (μM)
Dose-response curve for drug: Dasatinib
Inhibition (%)
Concentration (μM)
B
Dose-response matrix (inhibition)
10: 69.36 57.46 73.79 76.63 81.75 82.79
1.111: 46.42 54.79 52.24 70.16 78.03 77.66
0.123: 5.59 12.35 14.69 42.52 59.4 70.94
0.014: −0.52 0.89 8.18 36.93 52.56 66.12
0.002: −0.62 −1.17 3.24 26.27 38.3 59.57
0: −4.72 −4.14 4.57 21.91 35.66 55.8
AZD2014 (μM)
Dasatinib (μM)
inhibition (%)
C
HSA synergy score: 9.541
Loewe synergy score: 31.82
Bliss synergy score: 5.229
ZIP synergy score: 5.485
AZD2014 (μM)
Dasatinib (μM)
δ - score

Supplementary Figure S6. Quantification of AZD2014 and dasatinib synergy in S3 *NF2*-null SC-CRISPR cells. S3 cells were treated with the drugs for 72h using a 6x6 dose-response matrix, composing 5 drug concentrations ranging from 0.002 to 10 μM (1:9 serial dilution) and DMSO (vehicle) as a negative control. Synergistic response was determined by quantifying ATP levels which reflect metabolic activity using CellTiter-Glo viability kit. ATP levels were normalized to DMSO treatment and the % inhibition of metabolic activity was calculated relative to DMSO-treated cells. **A.** Dose-response curves of inhibition of metabolic activity as a function of different drug concentrations. **B.** Dose-response matrix heatmap. **C.** Synergy plots of AZD2014 and dasatinib combination matrices. 2D and 3D synergy maps illustrate synergistic dose regions (highlighted in red). Synergy score (δ-score) higher than 5 was considered indicative of true synergistic drug combination. Plots were created using the SynergyFinder web application for analyzing drug combination dose–response matrix data[3].

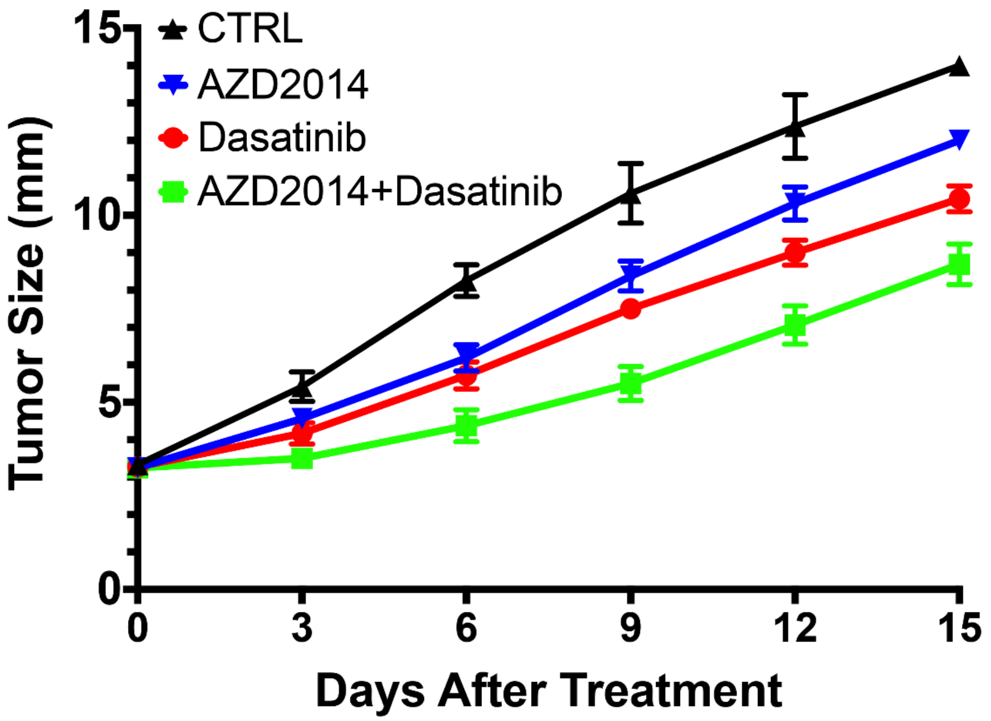

Supplementary Figure S7. Growth curve of *Nf2*$^{-/-}$ tumor in the mouse schwannoma model, comparing tumor growth associated with vehicle treated mice (n=13); mice treated with AZD2014 alone (15 mg/kg, n-16); mice treated with dasatinib alone (15 mg/kg, n=16); and mice treated with a combination of AZD2014 and dasatinib (n=14).

Supplementary References

1 Angus, S. P. *et al.* EPH receptor signaling as a novel therapeutic target in NF2-deficient meningioma. *Neuro Oncol.* **20**, 1185-1196 (2018).

2 Chou, T. C. & Martin, N. CompuSyn for drug combinations. pc software and user's guide. ComboSyn Inc Paramus NJ. (2005).

3 Ianevski, A., He, L., Aittokallio, T. & Tang, J. SynergyFinder: a web application for analyzing drug combination dose-response matrix data. *Bioinformatics.* **33**, 2413-2415 (2017).
